# Mental and physical health, impulse control, and intimate partner violence within a veteran sample

**DOI:** 10.3389/fpsyg.2025.1661296

**Published:** 2025-11-06

**Authors:** Hannah L. Grigorian, Helen P. Hailes, Kathleen M. Palm Reed, Erin D. Reilly

**Affiliations:** 1 VA Bedford Healthcare System, Bedford, MA, United States; 2 Kendall Psychological Associates, Cambridge, MA, United States; 3 Clark University, Worcester, MA, United States; 4 University of Massachusetts Chan Medical School, Worcester, MA, United States

**Keywords:** intimate partner violence, impulse control difficulties, PTSD-post traumatic stress disorder, chronic pain, insomnia

## Abstract

**Introduction:**

Psychiatric (e.g., PTSD, alcohol use disorder) and physical issues (e.g., chronic pain, sleep problems) are robustly associated with the use of intimate partner violence (IPV). These chronic conditions can amplify the likelihood of IPV use by increasing perceived threat, poor relationship quality, and negative affect while simultaneously decreasing the ability to inhibit violent behavior. However, the research in this area has largely been examined in limited samples or by examining a single chronic condition above diagnostic cut-offs or specific dual diagnosis (e.g., PTSD and alcohol use disorder). Further, potential mechanisms of action such as impulse control difficulties are rarely included in analyses.

**Methods:**

The current study aimed to extend prior research by examining mental health issues, physical health conditions, and impulse control difficulties in a cross-sectional survey design with a final community-based sample of 251 Veterans (188 male).

**Results:**

At the bivariate level, overall, psychological, and physical IPV use were respectively and positively associated (*p* < 0.05) with PTSD symptoms, alcohol use, chronic pain, sleep problems and impulse control difficulties; sexual IPV use was positively associated with all of these risk factors as well, except chronic pain. Within multiple regression analyses, impulse control difficulties were positively associated with overall IPV use (β = 0.28, *t* = 2.39, *p* = 0.02), psychological IPV (β = 0.44, *t* = 4.06, *p* < 0.001), physical IPV (β = 0.40, *t* = 3.39, *p* < 0.001), and sexual IPV (β = 0.40, *t* = 3.33, *p* = 0.001), even when controlling common demographic predictors (e.g., age, gender, income, social desirability) and in the context of other diagnostic risk factors (e.g., mental and physical health symptoms).

**Discussion:**

Findings suggest that difficulty inhibiting behavior when experiencing negative affect may be an important factor for IPV use in the context of multiple common psychiatric and physical issues. This potential area for intervention should be thoroughly examined in longitudinal and experimental designs.

## Introduction

The use of intimate partner violence (IPV; i.e., perpetration) is widely considered to be a global health concern spanning a large range of acts including psychological (e.g., threats of violence), physical (e.g., punching or slapping), and sexual violence (e.g., forced sexual contact or coercion; (Breiding et al., 2015). Understanding the primary contributing factors in the use of violence remains a key focus of IPV research, and mental and physical health problems present a promising avenue for predicting the likelihood of using violence. In meta analytic research, IPV use positively associated with mental health conditions across type including mood, personality, and trauma and stressor related disorders (Spencer et al., 2024). Of course, not all individuals with mental and physical health concerns use IPV, and the relative impact of mental and physical health, as well as potential mechanisms that may predict IPV use, remains understudied. One population that may particularly benefit from this research is military veterans who often present with a high prevalence of both IPV use and health problems (Kwan et al., 2020). However, veteran populations remain understudied in the IPV literature specifically within mental health correlates of IPV (Trevillion et al., 2015).

Following discharge from the military, veterans can encounter distinct psychosocial relationship challenges such as loss of purpose, identity confusion, and disconnection from civilian loved ones (Romaniuk and Kidd, 2018) as well as consequences related to their service that further exacerbate relationships (e.g., geographic mobility, service-related injuries, mental health problems). These challenges can increase difficulty reintegrating into family life and heighten interpersonal conflict with partners and IPV use (Sayers et al., 2009). Given these issues with community reintegration, it is perhaps not surprising that veterans also report worse overall mental health and more adverse mental health days compared to their civilian counterparts (Hoglund and Schwartz, 2014). Indeed, research suggests 63-92% of veterans have reported psychological, 26% physical, and 12–40% sexual IPV use in the past year (Kwan et al., 2020).

However, while these IPV rates speak to the veteran population overall, it is increasingly important to address gender differences in the use of IPV regarding prevalence rates, salient risk factors, and relative impact of IPV use. While psychological IPV use appears relatively consistent across binary gender, in military populations, men report higher rates of physical IPV use (27%) vs. women (22%) in recent meta analyses (Kwan et al., 2020). Additionally, the gender specific impact of IPV remains disproportionate, with a higher rate of injury for partners when physical IPV is used by military samples of men (Kwan et al., 2020). Relatedly, while any gender can sustain injury from IPV use, data from IPV-related violent deaths has shown that women are significantly more likely to be killed as a result of IPV use, while men are more likely to die by suicide, commit homicide and then die by suicide, or die by legal intervention (Caldwell et al., 2012; Kafka et al., 2021). Individual risk factors for IPV use such as common mental and physical health concerns also differ in strength depending on gender. For instance, while alcohol use remains a significant risk factor for IPV use across gender, it presents as a stronger risk factor for men (Spencer et al., 2022). As such, consideration of gender differences within theory-driven research examining how mental and physical health concerns correlate with IPV use overall and by type among veterans is needed.

Many common physical and mental health conditions appear to be correlated with IPV use among veterans (e.g., LaMotte et al., 2017; Martin et al., 2010; Crane and Easton, 2017). Specifically, healthcare utilization research has demonstrated that veterans who use IPV are most likely to access Veterans Health Administration (VHA) services for symptoms related to PTSD, chronic pain, sleep problems (e.g., insomnia), and physical conditions related to alcohol use (e.g., liver problems; Relyea et al., 2023). These four conditions likewise translate into increased likelihood of IPV use among veterans: veterans endorsing PTSD symptoms above the clinical diagnostic cut-off, hazardous alcohol use, chronic pain, or sleep problems have all demonstrated increased likelihood of using IPV in the past year (LaMotte et al., 2017; Crane and Easton, 2017; Portnoy et al., 2023). This relationship between IPV use and these issues is particularly concerning given the elevated rates of these conditions among veterans vs. the general population (Lehavot et al., 2018; Taylor et al., 2024; Panza et al., 2022; Grant et al., 2015; Nie et al., 2024; Bai et al., 2023).

Although use of medical record data and diagnosis-based predictive models for IPV use is vital, less is known about how symptom-specific and sub-clinical levels of these conditions, as well as managing multiple different conditions, may impact IPV use. For instance, when PTSD and alcohol use were considered together in a logistic regression in a veteran sample, only drinking days were associated with recent aggression, suggesting some conditions may contribute more variance to aggression than others when considered together (Flanagan et al., 2014). Findings such as these emphasize the importance of replicating and extending existing research through the consideration of multiple conditions in a single model to begin to parse their nuanced relationships to IPV. This is particularly important for the increasing amount of work extending this line of inquiry into understudied physical health disorders like sleep and chronic pain in IPV research, as both conditions have shown strong preliminary support for their influence on IPV use in the context of PTSD (LaMotte et al., 2017). Given the paucity of research, a theory-driven approach to hypothesizing the relationships between multiple conditions and IPV use is particularly indicated.

I3 theory, which defines violence risk as a combination of (1) instigating, (2) impelling, and (3) inhibiting factors, provides a helpful framework to conceptualize the impact of mental and physical health risk factors on IPV use (Finkel and Hall, 2018). (1) Instigation occurs when someone is exposed to a situation that can elicit aggression as a behavioral response option. For instance, the perception of an immediate threat to safety may afford an aggressive behavioral response. This area of risk can be heightened by increasing the frequency of threatening events (e.g., conflict in a relationship) or the mis-attribution of events as threatening (e.g., perceiving a threat from a neutral stimulus). However, instigation does not operate alone, and violence risk is further amplified by impelling and instigating factors. (2) Impelling factors influence the psychological state a person is in when they experience instigation. They are not inherent to the instigating event but are instead the stable factors which impact vulnerability to use aggression. For instance, trait level anger may impact how intensely an individual responds to instigation. Therefore, instigating and impelling factors may work together to exacerbate the likelihood of violence. (3) Inhibiting factors can override or contribute to the likelihood of violence depending on their strength. Inhibiting factors can be impacted by low trait levels of self-control, the exhaustion of mental resources, or a biological impact on inhibition (e.g., the consumption of alcohol). Instigating, impelling, and inhibiting factors are highly interactive and may encompass state and trait level variables. Existing theoretical and empirical research are utilized to categorize risk factors within this model and, frequently within IPV literature, predictors of IPV use are conceptualized to operate across I3 risk factors (Massa et al., 2020), particularly since IPV often occurs as a dynamic cycle in which consequences can become subsequent predictors of violence and/or internalized as trait-like dispositions. Therefore, it is helpful to consider risk factors that operate across multiple areas of vulnerability or present with a particularly strong effect. For instance, mental and physical health conditions are comprised of different symptoms which aggregate to diagnostic criteria and, when taken together, these symptoms can represent areas of risk across I3 theory. Applied to empirically supported mental and physical health concerns associated with IPV use for veterans, I3 theory illustrates how multiple conditions may have transdiagnostic underpinnings that function across IPV use risk factors. PTSD, alcohol use, chronic pain, and sleep problems cause vulnerabilities across instigating, impelling, and disinhibiting factors. There are a number of possible ways in which these factors might interact to increase risk for the use of IPV. For example, alcohol use, sleep deprivation, and hypervigilance symptoms are associated with misinterpreting neutral stimuli as threatening, thereby increasing the propensity for aggression (Lanius et al., 2017; Giancola et al., 2011; Krizan and Herlache, 2016). Impelling factors present within these conditions such as experiencing low mood or affective dysregulation, which are common across these conditions, further increase the likelihood for aggression (Minkel et al., 2012; Turk et al., 2016). These individuals are then more likely to see threat (instigation), be impelled to violence given enduring states central to their physical and mental health conditions, and experience disinhibition which decreases the likelihood of non-aggressive conflict resolution. Individuals experiencing symptoms of PTSD, alcohol misuse, chronic pain, and sleep problems may also experience disinhibition whether through biological impairment (e.g., alcohol use or sleep deprivation) or self-regulatory fatigue (e.g., PTSD and chronic pain) which can interfere with their ability to effectively manage instigating and impelling factors (Solberg Nes et al., 2009; Sadeh et al., 2018).

As a result, veterans managing alcohol use, sleep problems, PTSD, and/or chronic pain may encounter more situations where they are inclined to use violence (instigating), experience vulnerability to aggressive responses (impelling), and have a lower capacity to inhibit their responses (disinhibiting). However, despite frequent endorsement of multiple mental and physical health problems and their overlap within prevalent theoretical models such as I3 theory, these conditions are rarely considered together. More research is needed to delineate how much variance is contributed by each condition in multiple regression models. Further, past research is limited by examination of risk factors across types of IPV. Large-scale meta-analyses define distinct mental and physical risk factors for physical vs. psychological IPV (Spencer et al., 2024, 2022) while sexual IPV is left largely unstudied. More research is needed to examine the distinct correlations of mental and physical health correlates of IPV across IPV subtypes.

Further extensions of previous IPV work should include examination of potential mechanisms. Per I3 theory, increased risk for IPV occurs when instigating and impelling factors are high while the ability to inhibit behavior is low. This combination of risk factors is referred to as a “perfect storm” for violence (Finkel and Hall, 2018). Within this “perfect storm,” inhibitory factors may provide insight into key mechanisms driving aggressive responses. Using the I3 framework, difficulties with emotion regulation would be considered a particularly critical disinhibiting factor that increases risk of aggression in the context of instigating and impelling factors. Researchers have described deficits within emotion regulation to include non-acceptance of negative emotions, difficulty with impulse control when distressed, limited access to effective regulation strategies, and deficits in emotional awareness and clarity (Gratz and Roemer, 2004). Individuals with poor emotion regulation are more likely to respond aggressively to perceived threats (i.e., instigating factors; Shorey et al., 2011; Watkins et al., 2016). A recent literature review on dating violence among college students found that emotion regulation deficits consistently predict both physical and psychological IPV in men and women (Neilson et al., 2023). Additionally, emotion regulation can moderate the link between negative affect and physical IPV; that is, when emotion regulation is low, negative affect is more strongly associated with IPV behaviors (Shorey et al., 2015).

While overall poor emotion regulation has been associated with an increased risk of IPV use, difficulties with impulse control, in particular, are especially relevant within veteran populations. Difficulties with impulse control refers to the inability to regulate or restrain behavior when experiencing negative emotions (Gratz and Roemer, 2004). Impulse control has been strongly linked to general aggression (Bresin, 2019), IPV use (Bresin et al., 2022), can mediate reactive aggression (Gagnon and Rochat, 2017), and has been positively associated with IPV use in both court-mandated cases (Grigorian et al., 2019) and university samples (Gildner et al., 2021). Veterans with poor impulse control in the face of negative emotions may be particularly likely to use IPV if they are feeling unwell from pain, sleep disturbance, and PTSD symptoms. For example, researchers have found that emotion regulation, as a broad construct, fully accounted for the relationship between PTSD and impulsive aggression in a veteran sample (Miles et al., 2016) and that men with more trauma exposures and higher negative urgency were more likely to use IPV (Gildner et al., 2021). Yet, impulse control difficulties specifically have rarely been considered as a risk factor for IPV use for veterans or included as a potential mechanism to explain the impact of chronic conditions such as chronic pain, alcohol use, sleep problems, and PTSD severity on IPV use among veterans.

The current study aimed to build on prior IPV use research by examining the association among mental health conditions, physical health problems, and impulse control difficulties in a community based, non-clinical veteran sample. The hypothesized model first defines the bivariate relationships between common mental and physical health concerns and impulse control difficulties and IPV use. Following this, we examine the relative contributions of PTSD, alcohol use, insomnia, and chronic pain on IPV use overall and by type. Finally, as PTSD is one of the most well-studied mental health correlates of IPV use and interventions within the VHA (Creech et al., 2018) and well outlined in I3 theory, we extended this current literature through the examination of impulse control difficulties as a potential moderator in the relationship between PTSD and IPV use. To advance this understanding of IPV use, we investigated the following hypotheses:

1. PTSD symptoms, chronic pain, alcohol use, sleep problems, and impulse control difficulties would positively associate with psychological, physical, and sexual IPV use, respectively.
2. The proposed IPV use model variables (i.e., PTSD symptom severity, chronic pain, alcohol use, sleep problems, and impulse control difficulties) will each have a distinctive, significant association with veteran reported use of psychological, physical, sexual IPV, and overall IPV use, when modeled alongside empirically-supported control correlates of IPV use (i.e., age, gender, income, and social desirability).
3. Impulse control difficulties will moderate the relationship between PTSD symptoms and veteran reported use of psychological, physical, sexual IPV, and overall IPV use, such that the relationship between PTSD symptoms and IPV use will be stronger for individuals demonstrating high levels of impulse control difficulties.

## Materials and methods

### Procedures

This study was approved by the Bedford VA Healthcare System Institutional Review Board and preregistered on the Open Science Framework (OSF) webpage (information removed during peer-review process) prior to data collection. This study employed an anonymous, cross-sectional online survey design. Qualtrics, an experience management company, was contracted to host the survey and recruit veterans and send the study survey electronically via the platform to a sample of veterans already registered with the Qualtrics company as part of their available survey panels. Qualtrics has been successfully implemented with veteran samples to assess a broad range of mental and physical health concerns as well as functioning variables (Reilly et al., 2022). Online panel data have demonstrated equitable quality to traditional recruitment and collection methods (Walter et al., 2019) and can be further improved with rigorous data inclusion screening procedures (Heffner et al., 2021).

Veterans interested in participating were directed to an information page and study screening questions. To be eligible for this study, participants were required to be over 18, a United States veteran, and have been in a relationship for at least 1 month in the past year. Given the disproportionate number of male vs. female veterans, this screener also functioned to assist with oversampling for women veterans by restricting participation to approximately 70% male/30% female participants. Participants were paid through a Qualtrics credit card rewards program.

A total of 645 participants entered the survey link, with 302/645 (46.8%) completing the survey. Of the 343 who were terminated prior to survey completion, 121/343 (35.3%) participants had not been in a romantic relationship in the past year, 113/343 (32.9%) were determined not to be veterans, 37/343 (10.8%) declined consent and did not participate in the survey, 37/343 (10.8%) were not allowed to enter the survey as a result of completed participant quotas (e.g., gender stratification), 7/343 (2%) did not meet the age requirement (18 years of age), 2 (1%) were not in a relationship long enough (minimum 1 month), and finally, 26/343 (7.6%) did not pass Qualtrics quality response evaluations related to security and speed checks (e.g., bots and duplicates). Of the 302 participants who screened into and completed the survey, 51 were removed during data cleaning for problem response behaviors such as: repeating the same text too often in open-ended questions, straight-lining behavior (selecting the same response over and over in different questions), gibberish or non-sensical responses to an open-ended question, egregiously profane responses, or other suspicious response patterns.

### Participants

The final sample included 251 veterans who identified primarily as male (*n* = 188 male) with 62 participants identifying as “female”, and 1 participant who identified as a transgender woman. Participants identified primarily as White/Caucasian (83.3%), heterosexual (95.2%), and married (72.5%), Participants within the sample ranged from 21 to 90 years old with a mean age of 61.90 (SD = 13.95) and a reported average of 8.28 (SD = 7.9) years of military service. Veterans reported the average length of their current or most recent relationship to be 25.71 (SD = 19.20) years and the majority were currently living with their partner (80.5%). Of the sample, 62% endorsed the use of at least one act of IPV of any type in the past year with 61% reporting psychological IPV, 25% physical, and 9% sexual IPV. Only 73 (29.1%) of the veteran sample reported that the VHA was their primary source of health care, suggesting that the majority of surveyed veterans were not connected at all, or not strongly connected, to the VA care system. See Table 1 for extended demographic information detailing the sample.

**Table 1:** Sample demographics (*N* = 251).

**Variable**	**Frequency**	**Variable**	**Frequency**
**Gender**		**Income**	
Male	188 (74.9%)	Less than $19,999	11 (4.4)
Female	62 (24.7%)	$20,000–$39,999	33 (13.1%)
Transgender female/trans woman/MTF	1 (.4%)	$40,000–$59,000	57 (22.7%)
**Race** ^*^		$60,000–$79,000	49 (19.5%)
White/Caucasian	218 (86.9%)	$80,000–$99,999	29 (11.6%)
Black/African American	28 (11.2%)	$100,000–$149,999	46 (18.3%)
Native American (American Indian or Alaska Native)	8 (3.2%)	$150,000 +	26 (10.4%)
Asian American	3 (1.2%)	Relationship Status	
Multiracial	3 (1.2%)	Married	182 (72.5%)
A race not listed here	2 (.8%)	In a relationship, not married	25 (10%)
Declined to answer	1 (.4%)	Divorced, in a relationship	15 (6%)
**Ethnicity**		Divorced, single	13 (5.2%)
Not Spanish, Hispanic, or Latino	227 (90.4%)	Single, never married	12 (4.8%)
Spanish, Hispanic, or Latino	24 (9.6%)	Widowed, single	2 (0.8)
**Sexual orientation**		Widowed, in a relationship	2 (0.8)
Heterosexual	239 (95.2%)	Branch of Service^*^	
Lesbian	5 (2%)	Army	111 (44.2%)
Gay	4 (1.6%)	Air Force	58 (23.1%)
Bisexual	3 (1.2%)	Navy	50 (19.9%)
**Service Era** ^*^		Marines	19 (7.6%)
September 2001 or later	62 (24.7%)	National Guard	16 (6.4%)
August 1990-August 2001	89 (35%)	Army Reserves	8 (3.2%)
May 1975 – July 1990	86 (34.4%)	National Reserve	4 (1.6%)
Vietnam Era (1964-1975)	104 (41.4%)	Coast Guard	3 (1.2%)
February 1955-July 1964	9 (3.6%)	VHA as primary source of care	73 (29.1%)
Korean War (1950–1955)	1 (0.4%)		

^*^Participants could choose multiple categories.

### Measures

#### Demographics

Demographic assessment included questions related to, gender, race, ethnicity, sexual orientation, service era, income, relationship status, and branch of service.

#### IPV use

The Revised Conflict Tactics Scales 2 Revised Short Form (CTS-2 SF) measures the self-reported endorsement of acts of IPV across distinct subtypes of IPV: psychological aggression, physical assault, and sexual coercion over the past 12 months (Straus and Sugarman, 1996; Strauss and Warren, 2003). A dichotomous total score was calculated to represent the presence of any form of IPV use in the past year (1 = IPV endorsed, 2 = no IPV endorsement). Additionally, the psychological aggression, physical assault, and sexual coercion subscales of the CTS-2 SF were used to calculate past year frequency scores for the separate types of violence. There are 27 items total, 8 items in the psychological aggression subscale, 12 in the physical assault subscale, and 7 in the sexual coercion subscale. Items are rated on a 0 to 7 scale representing count variables, and a score of 0 indicates that the behavior has not occurred and 6 represents more than 20 instances of that behavior. Participants choose “7” if the behavior occurred outside of the past year. To calculate annual frequency of IPV acts per type, responses of “7” were recoded to “0” and scores 1–6 were recoded to represent the midpoint of endorsed acts within that range (1 = 1, 2 = 2, 3 = 4, 4 = 8, 5 = 15, 6 = 25). This scoring method for annual frequency and overall IPV use is recommended by scale developers (Straus, 2004b). Good reliability for psychological aggression (α = 0.74) and for physical assault (α = 0.88) has been demonstrated across cultural samples (Straus, 2004a). Internal consistency for the current study was acceptable for the psychological aggression (α = 0.69). Internal consistency was below the acceptable range for physical assault (α = 0.46), and sexual coercion (α = 0.23) scales. As IPV represents a behavioral index with low endorsement overall, it is typical for internal consistencies for the CTS-2 to cover a wide range and still be utilized as a gold standard within interpretation of research findings (Chapman and Gillespie, 2019).

#### PTSD symptoms

The Primary Care-PTSD (PC-PTSD) Screen (Prins et al., 2016) is a 5-item screening measure used to identify individuals with probable PTSD. This measure assesses exposure to a traumatic event as well as endorsement of past month PTSD symptoms (intrusive thoughts/re-experiencing, avoidance, cognitions/mood, and arousal/reactivity). Participants were first asked to endorse the presence of a criterion A trauma, and then responded to each symptom as yes (1) or no (0), with total scores range from zero to five. Good test-retest reliability (*r* = 0.83) has been established for this measure (Prins et al., 2003) and internal consistency was acceptable in the current study (α = 0.87).

#### Alcohol use

The Alcohol Use Disorder Identification Test (AUDIT-C; Bush et al., 1998) assesses alcohol use in the past year. This scale has 3 items with the scale ranging from 0 to 4 and is summed to create total scores ranging from 0 to 12. In prior research, the AUDIT-C was found to have good internal (α = 0.76) and test retest (*r* = 0.91) reliability (Barry et al., 2015; Jeong et al., 2017). Acceptable internal reliability was demonstrated for this study (α = 0.76).

#### Chronic pain

The Graded Chronic Pain Scale (GCPS-R; Von Korff et al., 2020) was designed to differentiate between mild, moderate, and severe levels of chronic pain. Six items were used to assess pain intensity and interference over the past 3 months. A combination of endorsement in intensity and interference determined the severity rating as presented in the validation article (Von Korff et al., 2020). While reliability was not examined in initial validation studies, the GCPS-R is based heavily on the three item Pain, Enjoyment, and General Activity Scale (PEG-3) which has evidenced acceptable reliability (α = 0.73–0.89) in past studies (Krebs et al., 2009) and high internal reliability in this study (α = 0.91). GCPS-R total score categorizations range from Grade 0 (chronic pain absent) to Grade 3 (high impact of chronic pain).

#### Sleep problems

The Insomnia Severity Index (ISI; Morin, 1993) is a 7-item measure of sleep impairment and distress consistent with insomnia in the past 2 weeks. Each item is scored on a 0 to 4 scale and summed to a total score ranging from 0 to 21, with higher scores indicating higher levels of sleep disturbance. Items assess a range of perceived sleep problems including severity (0 = none to 4 = very severe), satisfaction (0 = satisfied to 4 = very dissatisfied), noticeability (0 = not at all noticeable to 4 = very much noticeable), worry/distress (0 = not at all worried to 4 = very much worried), and functional interference (0 = not at all interfering to 4 = much interfering). Meta analyses and systematic reviews of studies using the ISI have indicated good internal reliability of the measure, with Cronbach's alpha at .80 or above (Manzar et al., 2021) which was consistent with the current study (α = 0.93).

#### Impulse control difficulties

The Impulse Control Difficulties subscale of the Difficulties in Emotion Regulation-Short Form Scale (DERS-SF; (Kaufman et al., 2016) measures one's ability to identify emotions, select relevant modulation strategies, and implement these strategies successfully. This subscale contains 3 items scored on a 1 (almost never or 0–10%) to 5 (almost always or 91–100%) scale. Scores were summed, with higher scores representing more difficulties with impulse control when experiencing negative emotions. Cronbach's alpha for the impulse control difficulties subscale was found to be 0.73 in the current study.

#### Social desirability

The Brief Social Desirability Scale (BSDS; Haghighat, 2007) is a 4-item measure used to assess socially desirable responding patterns, and is often used in survey research as a covariate to investigate potential for respondents to show reluctance in reporting socially objectionable or potentially illegal activity, which can lead to both over- or underreporting certain behaviors (Krumpal, 2013). Each question was answered in a yes/no format (yes = 1, no = 0), With higher scores indicating higher levels of impression management. Reliability for this measure was found to be adequate in its validation study (α = 0.60).

## Results

### Preliminary data analysis

All study variables were examined for assumptions of normality. Significant skew or kurtosis was determined as values greater than 2 or less than −2 by George, 2011) guidelines. The following variables violated this benchmark: the impulse control difficulties subscale of the DERS and the 3 subscales of the CTS-SF (psychological aggression, physical assault, and sexual coercion). All affected variables were log transformed. As IPV scales include the possibility of 0 (no acts in the past year), a constant of 1 was added to the total score prior to log-transformation. All subsequent study analyses used log transformed values. See Table 2 for skew and kurtosis before and after log-transformation. Multicollinearity was examined between study (chronic pain symptoms, impulse control difficulties, alcohol use, sleep problems, and PTSD symptoms). Study variables were found to have moderate inter-correlations with variance inflation factors (VIFs) within acceptable ranges (1 < VIF < 5; Belsley, 1993).

**Table 2:** Correcting for skew and kurtosis with log transformation.

**Variable**	**Skew (SE)**	**Kurtosis (SE)**
Psychological aggression	4.10 (0.15)	19.39 (0.31)
Psychological aggression (log transformed)	0.79 (0.15)	−0.12 (0.31)
Physical assault	8.45 (0.15)	83.19 (0.31)
Physical assault (log transformed)	4.05 (0.15)	17.81 (0.31)
Sexual coercion	4.40 (0.16)	19.05 (0.31)
Sexual coercion (log transformed)	3.53 (0.16)	11.28 (0.31)
DERS-impulse control difficulties	1.82 (0.15)	3.43 (0.31)
DERS-impulse control difficulties (log transformed)	0.83 (0.15)	0.04 (0.31)

Table 3 presents means and standard deviations for all study variables.

Prior to hypothesis testing, the relationship between study variables (i.e., psychological aggression, physical assault, sexual coercion, chronic pain symptoms, impulse control difficulties, alcohol use, sleep problems, and PTSD symptoms) and empirically supported sociodemographic control variables such as age, gender, household income, and social desirability were analyzed. In examining potential control variables, age, gender, household income, and social desirability were each associated with at least one study variable. Age was negatively associated with psychological aggression, sexual coercion, chronic pain, impulse control difficulties, alcohol use, sleep problems, and PTSD symptoms but not associated with physical assault. Household income was negatively associated with chronic pain, impulse control difficulties, alcohol use, sleep problems, and PTSD symptoms and not associated with psychological aggression, physical assault, or sexual coercion. Social desirability was negatively associated with impulse control difficulties and was not associated with other study variables. Gender was positively associated with psychological aggression, chronic pain, and sleep problems and not associated with physical assault, sexual coercion, impulse control difficulties, or PTSD symptoms. See Table 3 for bivariate associations as well as study descriptives. Given these findings, gender, age, household income, and social desirability were included as control variables within regression analyses.

**Table 3:** Bivariate correlations and descriptive statistics for study variables.

**Variable**	**1**	**2**	**3**	**4**	**5**	**6**	**7**	**8**	**9**	**10**	**11**	**12**	**13**
Overall IPV use	-												
Psychological IPV	0.78^**^	-											
Physical IPV	0.23^**^	0.47^**^	-										
Sexual IPV	0.23^**^	0.27^**^	0.32^**^	-									
Gender	0.16^*^	0.13^*^	0.08	−0.05	-								
Age	−0.18^**^	−0.19^**^	−0.08	−0.21^**^	−0.20^**^	-							
Income	−0.12	−0.07	−0.06	0.05	−0.03	0.15^*^	-						
Social desirability	0.03	−0.01	0.11	0.09	−0.01	0.14^*^	0.04	-					
Chronic pain	0.16^*^	0.27^**^	0.17^**^	0.04	0.15^*^	−0.13^*^	−0.10	0.01	-				
Impulse control difficulties	0.33^**^	0.52^**^	0.35^**^	0.30^**^	0.05	−0.35^**^	−0.14^*^	−0.14^*^	0.41^**^	-			
Alcohol use	0.16^*^	0.28^**^	0.26^**^	0.25^**^	0.25^**^	−0.22^**^	−0.04	−0.01	0.12	0.24^**^	-		
Sleep problems	0.29^**^	0.45^**^	0.29^**^	0.24^**^	0.23^**^	−0.41^**^	0.23	−0.05	0.48^**^	0.65^**^	0.30^**^	-	
PTSD symptoms	0.18^*^	0.35^**^	0.25^**^	0.19^*^	0.12	−0.42^**^	−0.42^**^	−0.01	0.48^**^	0.58^**^	0.42^**^	0.69^**^	-
*Mean*	-	5.98	0.46	1.51	1.26	61.90	4.17	3.72	0.81	5.16	2.58	8.00	1.93
*(SD)*	-	(12.99)	(2.33)	(6.13)	(0.47)	(13.95)	(1.71)	(0.92)	(1.16)	(2.59)	(2.51)	(6.51)	(1.97)

^*^p < 0.05.

^**^p < 0.01 (two-tailed).

Means and standard deviations calculated from non-transformed variables.

### Hypotheses 1 and 2

Bivariate correlations were run to determine the preliminary associations between chronic pain, logged impulse control difficulties, alcohol use, sleep problems, and PTSD symptoms and 1) the occurrence of overall IPV use in the past year (Hypothesis 1) and 2) the reported annual frequency of logged psychological aggression, logged physical assault, and logged sexual coercion, respectively (Hypothesis 2). Overall IPV use was positively associated with higher levels of chronic pain, impulse control difficulties, alcohol use, sleep problems, and PTSD symptoms. Logged psychological aggression and logged physical assault were both positively associated with higher levels of chronic pain, logged impulse control difficulties, alcohol use, sleep problems, and PTSD symptoms. Logged sexual coercion was positively associated with impulse control difficulties, alcohol use, PTSD symptom severity, and sleep problems but not chronic pain, see Table 3.

### Hypothesis 3

Multiple linear regression analyses were used to test Hypothesis 3. We examined the relationship between and unique variance in past year IPV explained by higher levels of PTSD symptom severity, chronic pain intensity, sleep problems, and logged impulse control difficulties (Block 2) use while controlling for gender, age, household income, and social desirability (Block 1). Four separate regressions using this hierarchical model were conducted with the following distinct IPV outcomes: overall IPV use, logged psychological aggression, logged physical assault, and logged sexual coercion. In consideration of the increased family-wise error rate when including multiple independent variables in regressions (Perrett et al., 2006) a Bonferroni adjustment was used. Accounting for the four controls and the five study variables (chronic pain, alcohol use, sleep problems, and PTSD symptoms, and logged impulse control difficulties) an adjusted alpha level of 0.006 (0.05/9) was applied to determine significance.

#### Overall IPV use

The addition of chronic pain, alcohol use, sleep problems, PTSD symptoms, and impulse control difficulties (Block 2) to control variables (gender, age, household income, and social desirability (Block 1) explained a significant amount of variance in overall IPV use, *F*_(9, 127)_ = 3.01, *p* < 0.01, ΔR2 = 0.07. In consideration of the adjusted alpha level (*p* = 0.006), the logged impulse control difficulties subscale of the DERS (β = 0.28, *t* = 2.39, *p* = 0.02), chronic pain (β = −0.30, *t* = −0.29, *p* = 0.77), alcohol use (β = 0.03, *t* = 0.34, *p* = 0.74), sleep problems (β = 0.19, *t* = 1.37, *p* = 0.17), and PTSD symptoms (β = −0.20, *t* = −1.60, *p* = 0.11) were not significantly associated with overall IPV use. The entire model accounted for 12% of the variance in the overall IPV use variable (see Table 4 for estimates within the hierarchical regression analysis).

**Table 4:** Hierarchical regression: overall IPV use.

	**Variable**	***B (SE)***	**β**	***t***	***p***	***R^2^adj(SE)***	***ΔR2***
**Step 1**						0.05 (0.45)	
	Gender	0.12 (0.08)	0.13	1.50	0.14		
	Age	−0.00 (0.00)	−0.20	−2.25	0.026		
	Income	−0.03 (0.02)	−0.11	−1.25	0.21		
	Social desirability	0.02 (0.04)	0.03	0.40	0.69		
**Step 2**						0.12 (0.43)	0.07
	Gender	0.11 (0.08)	0.12	1.41	0.16		
	Age	−0.00 (0.00)	−0.11	−1.13	0.26		
	Income	−0.03 (0.02)	−0.09	−1.11	0.27		
	Social Desirability	0.03 (0.04)	0.07	0.83	0.41		
	Chronic Pain	−0.01 (0.04)	−0.30	−0.29	0.77		
	Impulse Control Difficulties	0.28 (0.12)	0.28	2.39	0.02		
	Alcohol Use	0.01 (0.01)	0.03	0.34	0.74		
	Sleep Problems	0.01 (0.01)	0.19	1.37	0.17		
	PTSD Symptoms	−0.05 (0.03)	−0.20	−1.60	0.11		

^*^Significance demonstrated within adjusted alpha level (*p* < 0.006).

#### Psychological aggression

A significant amount of variance was explained in past year logged psychological aggression by the addition of study variables (chronic pain, alcohol use, sleep problems, and PTSD symptoms) to controls (gender, age, household income, and social desirability), *F*_(9, 127)_ = 6.49, *p* < 0.001, ΔR2 = 0.26. Logged impulse control difficulties positively associated with past year logged psychological aggression (β = 0.44, *t* = 4.06, *p* < 0.001). All other study variables were not significant in the final model: chronic pain (β = −0.04, *t* = −0.45, *p* = 0.65), alcohol use (β = 0.08, *t* = 0.92, *p* = 0.36), sleep problems (β = 0.22, *t* = 1.74, *p* = 0.08), and PTSD symptoms (β = −0.05, *t* = −0.41, *p* = 0.68). The entire model accounted for 27% of the variance in the logged psychological aggression IPV use variable (see Table 5).

**Table 5:** Hierarchical regression: psychological aggression.

	**Variable**	***B (SE)***	**β**	***t***	***p***	***R^2^adj(SE)***	***ΔR2***
**Step 1**						0.00 (1.24)	
	Gender	0.08 (0.22)	0.03	0.39	0.70		
	Age	0.01(0.01)	−0.17	−1.85	0.07		
	Income	−0.01(0.06)	−0.01	−0.10	0.92		
	Social Desirability	0.00(0.11)	0.00	0.03	0.97		
**Step 2**						0.27 (1.06)	0.26
	Gender	0.08 (0.19)	0.03	0.41	0.68		
	Age	0.00 (0.01)	0.05	0.56	0.58		
	Income	0.04 (0.06)	0.05	0.67	0.50		
	Social desirability	0.06 (0.10)	0.05	0.58	0.56		
	Chronic pain	−0.04 (0.09)	−0.04	−0.45	0.65		
	Impulse control difficulties	1.17 (0.29)	0.44	4.06	< 0.001^*^		
	Alcohol use	0.03 (0.04)	0.08	0.92	0.36		
	Sleep problems	0.04 (0.02)	0.22	1.74	0.08		
	PTSD symptoms	−0.03 (0.07)	−0.05	−0.41	0.68		

^*^Significance demonstrated within adjusted alpha level (*p* < 0.006).

#### Physical assault

The addition of chronic pain, alcohol use, sleep problems, PTSD symptoms, and logged impulse control difficulties (Block 2) to control variables (gender, age, household income, and social desirability; Block 1) explained a significant amount of variance in physical assault, *F*_(9, 127)_ = 3.72, *p* < 0.001, ΔR2 = 0.15. Logged impulse control difficulties positively associated with past year physical assault (β = 0.40, *t* = 3.39, *p* < 0.001), above and beyond all other variables which were non-significant in the final model: chronic pain (β = −0.15, *t* = −1.57, *p* = 0.12), alcohol use (β = 0.15, *t* = 1.69, *p* = 0.09), sleep problems (β = 0.06, *t* = 0.41, *p* = 0.69), and PTSD symptoms (β = 0.03, *t* = 0.21, *p* = 0.83). The entire model accounted for 15% of the variance in the logged physical aggression IPV use variable (see Table 6).

**Table 6:** Hierarchical regression: physical assault.

	**Variable**	***B (SE)***	**β**	***t***	***p***	***R^2^adj(SE)***	***ΔR2***
**Step 1**						0.00 (0.58)	
	Gender	0.07 (0.10)	0.01	0.14	0.89		
	Age	0.01 (0.00)	−0.10	−1.13	0.26		
	Income	−0.00 (0.03)	−0.01	−0.09	0.93		
	Social Desirability	0.10 (0.05)	0.17	1.91	0.06		
**Step 2**						0.15 (0.54)	0.15
	Gender	0.06 (0.10)	0.06	0.65	0.52		
	Age	0.00 (0.00)	0.08	0.78	0.44		
	Income	0.02 (0.03)	0.05	0.54	0.59		
	Social desirability	0.12 (0.05)	0.21	2.46	0.02		
	Chronic pain	−0.07 (0.05)	−0.15	−1.57	0.12		
	Impulse control difficulties	0.49 (0.15)	0.40	3.39	< 0.001^*^		
	Alcohol use	0.03 (0.02)	0.15	1.69	0.09		
	Sleep problems	0.00 (0.01)	0.06	0.41	0.69		
	PTSD symptoms	0.01 (0.04)	0.03	0.21	0.83		

^*^Significance demonstrated within adjusted alpha level (*p* < 0.006).

#### Sexual coercion

A significant amount of variance was explained in past year sexual coercion by the addition of study variables (chronic pain, alcohol use, sleep problems, PTSD symptoms, and logged impulse control difficulties) to controls (gender, age, household income, and social desirability), *F*_(9, 125)_ = 3.98, *p* < 0.001, ΔR2 = 0.10. Logged impulse control difficulties positively associated with sexual coercion (β = 0.40, *t* = 3.33, *p* = 0.001) above and beyond all other study variables. Social desirability was the only other variable to enter the specified range of significance *(p* = 0.006) and positively associated with sexual coercion (β = 0.27, *t* = 3.16, *p* = 0.002). Chronic pain (β = −0.05, *t* = −0.46, *p* = 0.65), alcohol use (β = 0.16, *t* = 1.76, *p* = 0.08), sleep problems (β = −0.02, *t* = −0.14, *p* = 0.89), and PTSD symptoms (β = −0.10, *t* = −0.83, *p* = 0.41) did not associate with sexual coercion. The entire model accounted for 17% of the variance in the logged sexual coercion IPV use variable (see Table 7).

**Table 7:** Hierarchical regression: sexual coercion.

	**Variable**	***B (SE)***	**β**	***t***	***p***	***R^2^adj (SE)***	***ΔR2***
**Step 1**						0.07 (0.82)	
	Gender	−0.19 (0.14)	−0.11	−0.25	0.20		
	Age	−0.02 (0.01)	−0.25	−1.13	0.01^*^		
	Income	0.05 (0.04)	0.01	−0.10	0.24		
	Social desirability	0.18 (0.07)	0.21	2.48	0.01		
**Step 2**						0.17 (0.78)	0.10
	Gender	−1.07 (0.14)	−0.06	−0.75	0.46		
	Age	−0.01 (0.01)	0.08	−1.55	0.12		
	Income	0.07 (0.04)	0.13	1.66	0.10		
	Social desirability	0.23 (0.07)	0.27	3.16	0.002^*^		
	Chronic pain	−0.03 (0.07)	−0.05	−0.46	0.65		
	Impulse control difficulties	0.72 (0.22)	0.40	3.33	0.001^*^		
	Alcohol use	0.05 (0.03)	0.16	1.76	0.08		
	Sleep problems	−0.00 (0.02)	−0.02	−0.14	0.89		
	PTSD symptoms	−0.04 (0.05)	−0.10	−0.83	0.41		

^*^Significance demonstrated within adjusted alpha level (*p* < 0.006).

### Moderation analyses

Four multiple regression models were tested to investigate whether the association between PTSD symptoms and IPV use was related to the level of impulse control difficulties for veterans. PTSD and impulse control difficulties were both mean centered and then multiplied to create an interaction term. Block 1 included all control variables (age, gender, income), and Block 2 added mean centered PTSD and impulse control difficulties total scores, and block 3 further added the interaction term. Interaction terms were non-significant in models for overall IPV use (β = −0.04, SE = 0.04, *t* = −1.09, *p* = 0.28), psychological IPV use (β = 0.02, SE = 0.09, *t* = 0.25, *p* = 0.81), physical IPV use (β = 0.06, SE = 0.05, *t* = 1.3, *p* = 0.20), and sexual IPV use (β = 0.06, SE = 0.07, *t* = 0.90, *p* = 0.37).

## Discussion

The current study extends the current literature by investigating the I3 theoretical framework to further explore the relationship between multiple risk factors for IPV use in a community sample of veterans (Finkel, 2014). Military veterans may be at heightened risk of IPV use due to the frequent endorsement of multiple chronic conditions such as PTSD, alcohol misuse, chronic pain, and sleep disturbances—each strongly linked to IPV risk factors (Irizar et al., 2021; Folmer et al., 2020; Qureshi et al., 2023; Trivedi et al., 2015). While PTSD, alcohol misuse, chronic pain, and sleep problems are independently associated with IPV use in veteran samples (LaMotte et al., 2017; Martin et al., 2010; Crane and Easton, 2017), the reporting multiple conditions with symptoms across clinical thresholds and within unrestricted demographic samples (e.g., open recruitment to all service eras, genders, and marital statuses) remains understudied. Additionally, existing research examining chronic conditions rarely consider underlying mechanisms which may drive IPV use such as impulse control difficulties.

Consistent with Hypothesis 1, findings indicated that PTSD symptoms, alcohol use, chronic pain severity, sleep problems, and impulse control difficulties were positively associated with overall IPV use, psychological aggression, and physical assault in the past year. Findings from the current study indicate that increasing levels of these symptoms were associated with higher levels of IPV when considered continuously (i.e., not dichotomized at clinical thresholds). This extends the prior literature base which focused on the relationship between IPV and conditions that were diagnostic or based on screening cut-offs. With further corroborating research, these findings may indicate the utility of IPV prevention and intervention programs aimed at assisting veterans with multiple issues across a board range of symptom severity.

However, inconsistent with previous studies (e.g., Taft et al., 2010; Shorey et al., 2012) and Hypothesis 1 predictions, sexual coercion use was positively associated with all variables except chronic pain. While this finding is contrary to some existing research (Taft et al., 2010), there is a significant lack of literature examining sexual coercion and IPV use among veterans experiencing chronic pain. Specifically, the assessment and interpretation of sexual IPV is broadly underassessed and frequently excluded from IPV studies further warranting examination within this study. In fact, sexual IPV data are so limited and heterogenous in military samples that they are frequently unable to be analyzed within systematic or meta analyses (e.g., Kwan et al., 2020). Within chronic pain studies specifically, research typically only examines physical or psychological IPV (Spencer et al., 2024; Crane and Easton, 2017; Singh et al., 2014) or includes brief (e.g., 3-item; Taft et al., 2010) measures of sexual IPV use. The current study is novel in that it includes the sexual coercion subscale of the CTS-2 as a distinct outcome. While this finding could point to differences within IPV typology, replication is necessary to warrant full interpretation.

To test Hypothesis 2 and examine both the combined and unique impacts of our model variables, our study examined these risk factors in a single model with supported controls (i.e., age, gender, income, and social desirability) on different types of IPV use (overall, sexual, psychological, and physical). Although the full model explained a significant amount of the variance in IPV use overall, no condition-specific model variables (e.g., PTSD, insomnia symptom severity) were uniquely and significantly associated with overall IPV use. However, impulse control difficulties were associated with psychological aggression, physical assault, and sexual coercion above all other empirically supported risk-factors.

These findings further emphasize the vital importance of impulse control difficulties in the annual frequency of IPV use across psychological, physical, and sexual sub-types, above and beyond chronic condition symptom severity or the endorsement of multiple health difficulties. I3 theory emphasizes the importance of the relative strength of these factors, proposing that violence is most likely when instigating and impelling factors are high while inhibiting factors are low (Finkel, 2014). Experimental paradigms have revealed that IPV use is higher for participants with high provocation (instigation), impelling factors (trait anger), and inadequate inhibitory strategies such as attempts at thought suppression (Birkley and Eckhardt, 2019). However, impulse control difficulties as a mechanism of IPV use in the context of mental and physical health conditions should not be over-interpreted in the current study. Contrary to Hypothesis 3, impulse control difficulties were not found to moderate the association between PTSD symptoms and IPV (overall, psychological, physical, or sexual). While PTSD severity did not uniquely predict IPV use among individuals with poor impulse control compared to those with better impulse control, difficulties with impulse control did emerge as a significant predictor of IPV use across the full sample. This suggests that impulse control challenges may contribute to aggression and PTSD severity broadly, rather than being confined to specific high-risk groups. Notably, prior research has indicated that emotion regulation may fully explain the link between PTSD and impulsive aggression in veteran populations (Miles et al., 2016). This is contrary to current study findings which did not support impulse control difficulties as a moderator between PTSD symptoms and IPV and suggests further examination and replication.

## Limitations and areas for future research

Study findings and implications should be understood in the context of a number of limitations. First, this study utilized a cross-sectional design; further experimental and longitudinal studies are needed to delineate the possible causal role of impulse control difficulties on IPV use in the context of chronic clinical conditions. This is particularly important given the assessment timelines across measures utilized within the current study. While alcohol and IPV are assessed within the past year, PTSD, chronic pain, and insomnia measures utilize timelines ranging from the past 2 weeks to the past 30 days. Therefore, the current study is unable to make direct claims about the co-occurrence of symptoms. Temporal studies can better address this important area of IPV research. Another limitation of this study was the lack of nationally representative veteran sample. While we utilized a sample without clinical cut-offs or population restrictions, data are not nationally representative in terms of sociodemographic variables for the national veteran population. For instance, 87% of study participants identified as Caucasian in the current study compared to only 76% in the total veteran population (VetPop2023, 2023), limiting generalizability. Additionally, in consideration of participant burden and data quality, we utilized brief screeners to assess mental and physical health problems. The selection of these measures was directly informed by Veteran Affairs screening procedures and informed measures used for alcohol misuse, PTSD symptoms, and sleep problems (Prins et al., 2016; Bush et al., 1998; Morin, 1993). Therefore, wherever possible, measures representing typical screening procedures for veterans accessing VHA care were used. However, the brevity of measures also precludes comprehensive assessment of conditions or the examination of particular symptom profiles, an important area for future inquiry. This may be particularly helpful for PTSD in which certain symptom clusters like hyperarousal have been isolated as specific risk areas for IPV use (Birkley et al., 2016).

Findings about IPV subtypes should be considered in the context of psychometric limitations. Sexual IPV measured via the “sexual coercion” subscale of the CTS-2 demonstrated poor reliability and low endorsement. Given this, overinterpretation of these findings is cautioned against and future research should consider present limitations to improve data collection. It is probable that differences in sexual coercion findings reflect overall limitations in the measurement of sexual IPV, particularly self or partner reports such as the CTS-2SF (Straus and Sugarman, 1996; Strauss and Warren, 2003). This measurement issue presents as a common barrier within IPV literature. In consideration of the absence of sexual coercion data within extant literature examining chronic conditions and IPV use, other studies may have experienced similar reliability issues and foregone the use of sexual coercion data to preserve data quality. Future studies should discuss their choice for inclusion or exclusion of sexual coercion data and provide reliability data on subsequent measures for use in scoping analyses and reviews. When included, sexual coercion measures may benefit from more extensive measures of social desirability than used in the current study. Previous findings demonstrate that under-reporting “undesirable” sexual behavior is particularly vulnerable to the influence of social desirability within surveys (King, 2022). Future studies may consider the use of longer, widely use measures of social desirability (e.g., Crowne and Marlowe, 1960) to better examine impact and facilitate comparisons across studies.

Future studies may also wish to expand the investigation of IPV and chronic conditions in veteran populations to additional, population-specific risk factors such as head injuries including traumatic brain injuries (TBIs). According to the National Academy of Sciences (Institute of Medicine, 2013), symptoms of TBIs can include problems with executive functioning, mood, physical impairment, and social dysfunction. These symptoms may operate across areas of IPV risk; for example, within longitudinal veteran samples, persistent post-concussion symptoms were found to predict IPV use above and beyond controls including binge drinking, pain severity, and PTSD (Portnoy et al., 2022). Examining head injury in the context of other chronic conditions and underlying risk factors (e.g., impulse control difficulties) would build on the current study and existing research to better define the relative importance of these factors on the likelihood of IPV use.

Future research should continue to integrate multiple risk and protective factors of IPV use within a cohesive theoretical framework rather than examining them in isolation. The I3 theory provides a valuable lens for understanding how predictors both interact and operate independently to heighten the risk of IPV use. Increasingly, research has highlighted emotion dysregulation as a key disinhibiting process linked to both PTSD (Seligowski et al., 2015; McLean and Foa, 2017) and IPV use (Maloney et al., 2022). Further investigation is needed to clarify which aspects of emotion dysregulation contribute most to poor mental health and aggression. For instance, research suggests that deficits in emotion regulation strategies, emotional clarity, and emotional acceptance are particularly associated with PTSD severity (Christ et al., 2021). Consequently, these may be prime targets for not only additional measurement in research, but also areas to target in future clinical interventions.

## Conclusions

Findings suggest that difficulty inhibiting behavior when experiencing negative affect may be a driving factor for IPV use above and beyond the presence of multiple, common mental and physical health issues. Study findings should be understood in the context of limitations related to cross-sectional design, social desirability, and psychometric concerns. The role of impulse control on IPV use risk in veterans should be thoroughly examined in longitudinal and experimental designs, using nationally representative samples, and in consideration of related constructs (e.g., TBIs). Given the scarcity of research on underlying mechanisms of IPV use in veterans, as well as tailored intervention options for veterans who use IPV, additional research is needed to further delineate veteran-specific risk and predictive factors for IPV informed by the findings and limitations of the current study.

## Data Availability

The datasets generated and analyzed during this study are not publicly available due to the security requirements of the Department of Veterans Affairs but are available from the corresponding author on reasonable request. The authors will consider reasonable requests on a case-by-case basis, subject to compliance with the Department of Veterans Affairs data sharing agreements. Requests to access the datasets should be directed to Hannah Grigorian, hannah.grigorian@va.gov.
